# Climate Transformation and Stewardship: Reflections on Meaningful Collaboration to Support Indigenous‐Led Research

**DOI:** 10.1002/ece3.72715

**Published:** 2025-12-22

**Authors:** Megan K. Jennings, Amber Pairis, Althea Walker, William Madrigal, Diane Terry, Joelene Tamm, Connor Magee, Alexandra Hoff, M. Brooke Rose, Gregory A. Backus, Clarissa Rodriguez, Lluvia Flores‐Renteria, Janet Franklin, Helen M. Regan

**Affiliations:** ^1^ Department of Biology San Diego State University San Diego California USA; ^2^ Climate Science Alliance San Diego California USA; ^3^ Desert Research Institute Reno Nevada USA; ^4^ Department of Anthropology University of California Riverside California USA; ^5^ La Jolla Band of Luiseño Indians Pauma Valley California USA; ^6^ Graduate Group in Ecology University of California Davis California USA; ^7^ Center of Open Geographical Sciences, Department of Geography San Diego State University San Diego California USA; ^8^ Department of Evolution, Ecology, and Organismal Biology University of California Riverside California USA

**Keywords:** climate change adaptation, climate resilience, ecological restoration, Indigenous knowledges, Indigenous‐led research, terrestrial plant communities

## Abstract

We are a group of non‐Indigenous and Indigenous scientists, culture bearers, and natural resource managers who came together with the aid of a boundary‐spanning organization to conduct research to support climate‐resilient ecological and cultural restoration carried out by the Tribal communities of southern California. Climate change and other environmental harms caused by human industrial activities disproportionately burden communities that have contributed the least to the problem. Addressing the environmental injustices wrought by climate change requires relationships between Indigenous peoples and other entities in society that are built on consent, trust, accountability, and reciprocity—qualities that are historically lacking in Western academic institutions. Therefore, co‐designed and co‐led research with Indigenous communities is desired to achieve equal collaboration in building deeper environmental understanding and implementing management strategies that reflect shared priorities between Western conservation agendas and Tribal Nations. In this paper, we share foundational principles for meaningful engagement and implementing community‐led climate adaptation planning that centers Tribal community partners and the core tenets of respect, responsibility, reciprocity, and relationships. We describe our project aimed at advancing a model for Indigenous‐led climate adaptation in Southern California, USA, focused on restoring resilient ecological communities, and reflect on lessons learned by researchers and environmental stewards about co‐creation of climate futures from this collaboration. These reflections inform and improve our collaborative framework so that it can be applied more widely.

## Graphical abstract

This paper describes a project aimed at advancing a model for Indigenous‐led climate adaptation in Southern California, USA, focused on restoring resilient ecological communities, and reflect on lessons learned by researchers and environmental stewards about coproduction of climate futures from this collaboration. We share foundational principles for meaningful engagement and implementing community‐led climate adaptation planning that centers Tribal community partners and the core tenets of respect, responsibility, reciprocity, and relationships.

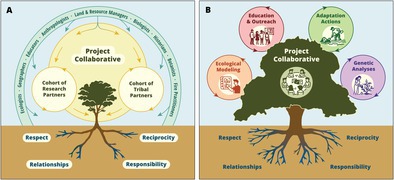

## 1 Introduction

The long‐predicted impacts of climate change are irreversibly playing out at local, regional, and global scales (IPCC 2021). As a result, wildfires, drought, extreme weather events, and prolonged periods of heat are threatening human and natural communities. Climate change is also pressuring biological diversity, with ~17% of animal and plant species predicted to be lost in the next 50 years (Wiens and Zelinka 2024). It is well established that interdisciplinary, cross‐sectoral approaches to planning are needed to advance climate adaptation to address current and future impacts. However, recent studies have highlighted a disconnect between planning and implementation (Dovers and Hezri 2010; Bierbaum and Stults 2013; Shi and Moser 2021). In particular, relevant science (Singh et al. 2020) and expertise (Dannevig et al. 2012) are needed to advance climate adaptation planning and implementation. Collaborative development of regionally specific approaches, where partners can share scientific and place‐based knowledges, resources, and strategies to achieve on‐the‐ground results, can be critical to meeting the challenges of a rapidly changing climate. The question remains how to appropriately and equitably connect with community partners to ensure that research is relevant and reflects the priorities and needs of those it is intended to serve.

Climate change and other environmental harms caused by human industrial activities disproportionately burden communities that have contributed the least to the problem (Füssel 2010; Robinson 2018; Gonzalez 2020). Native American communities and Indigenous populations worldwide that have long been subjected to environmental injustices and truly existential threats—colonization, genocide, forced migration, land dispossession, enslavement (Tuhiwai Smith 2012)—are experiencing the impacts of climate change in ways that deeply affect their communities, cultures, and lifeways. Moreover, “fortress” or “colonial” biodiversity conservation actions through the creation of protected areas “where ecosystems can function in isolation from human disturbance” have historically dispossessed local Indigenous communities from their homelands causing further injustice (Plumwood 2012; Domínguez and Luoma 2020). Hence, research projects that focus on Indigenous partners but lack a commitment to responsible, meaningful partnership can do harm and jeopardize future opportunities to collaborate.

Don Hankins, a traditional cultural practitioner, Miwkoʔ language speaker, and Professor of Geography and Planning at California State University, Chico, recently advocated for climate resilience through Indigenous ecological stewardship in California, USA, positing that ecological stewardship *is* cultural stewardship and that stewardship is an intergenerational lifeway responsibility (Hankins 2024). Healthy ecosystems support healthy communities and reflect Indigenous relationships to place through land, waters, and beyond. Hankins argued that Indigenous communities often recognize colonialism as the beginning of the climate crisis. As such, decolonizing our collective efforts to respond to climate change is critical. Addressing the environmental injustices wrought by climate change on Indigenous peoples requires relationships between Indigenous peoples and other entities in society that are built on consent, trust, accountability, and reciprocity—qualities that are historically lacking (Whyte 2017) in Western academic institutions. There is an opportunity and a need for new ways of addressing the climate crisis and achieving climate and environmental justice through Indigenous‐led ecological research (Bartlett et al. 2012), climate adaptation and ecological restoration (e.g., Thoreson et al. 2024; Mooney‐D'Arcy 2013).

Conservation and restoration of natural ecosystems is an important tool for both climate change adaptation and mitigation, falling under the framework of nature‐based solutions (Seddon et al. 2020; McElwee et al. 2023). Ecological restoration is defined as “the process of assisting the recovery of an ecosystem that has been degraded, damaged or destroyed” (Martin 2017). It has recently been recognized that ecosystem restoration can play a role in personal and social transformation (psychosocial resilience; Smith et al. 2025); those authors, however, did not focus on Indigenous communities. Rather, they borrowed the concept of reciprocity to add to the Western scientific understanding of restoration—an example of assigning supplemental value of Indigenous knowledges to the dominant culture (*sensu* Whyte 2017).

There have been calls for building cultural values into restoration, and a global survey showed that successful projects actively involved Indigenous peoples in co‐designing restoration activities affecting their territories (Reyes‐García et al. 2019). In Australia, there is increasing recognition of the value of involving Indigenous peoples in conservation, and ecological research can play a role in reinforcing the practices of Indigenous stewardship (Barbour and Schlesinger 2012). However, challenges remain where Indigenous partners “do not want to become spectators in the research process, giving away knowledge, or laborers to Western conservation agendas. They want to be active partners in developing better understandings of the environment and implementers of management that reflects shared agendas” (Barbour and Schlesinger 2012, 36).

In this paper we present a Tribally led effort to promote ecocultural resilience in Southern California by bringing an Indigenous worldview into academic research that effectively supports climate adaptation. An Indigenous worldview (“Indigenous knowledges”) conceives a relationship between humans and nature—one of kinship and reciprocity—that is different from the dominant culture's commodification and objectification of nature (Kimmerer 2013). “[N]atural relatives (not resources) are gifts from the earth…” (Nelson 2018, 256). Indigenous worldviews also value responsibility to the (human and non‐human) community and reciprocity between generations—responsibility to ancestors and descendants (e.g., Kimmerer 2018). The goal of our research was to develop knowledges and support actions that enhance persistence of Indigenous ecological and cultural stewardship practices through a process that is visioned, led, and implemented by the communities it serves. The “Resilient Restoration” project was designed to advance understanding of the impacts of climate change on a range of native plant species that serve as the foundation of Southern California's biodiversity and are critical to Tribal cultures, health, and well‐being. In this paper we share core concepts for meaningful engagement and implementing community‐led climate adaptation planning that centers Tribal community partners (Section 2). We describe our project aimed at advancing a model for Indigenous‐led climate adaptation in southern California focused on restoring resilient ecological communities (Section 3) and reflect on lessons learned by researchers and environmental stewards about coproduction of climate futures from this collaboration (Sections 3.1, 3.4). These reflections inform and improve our collaborative framework so that it can be applied more widely (Section 4).

## 2 Foundations of Respect, Responsibility, Reciprocity, and Relationships

**FIGURE 1:**
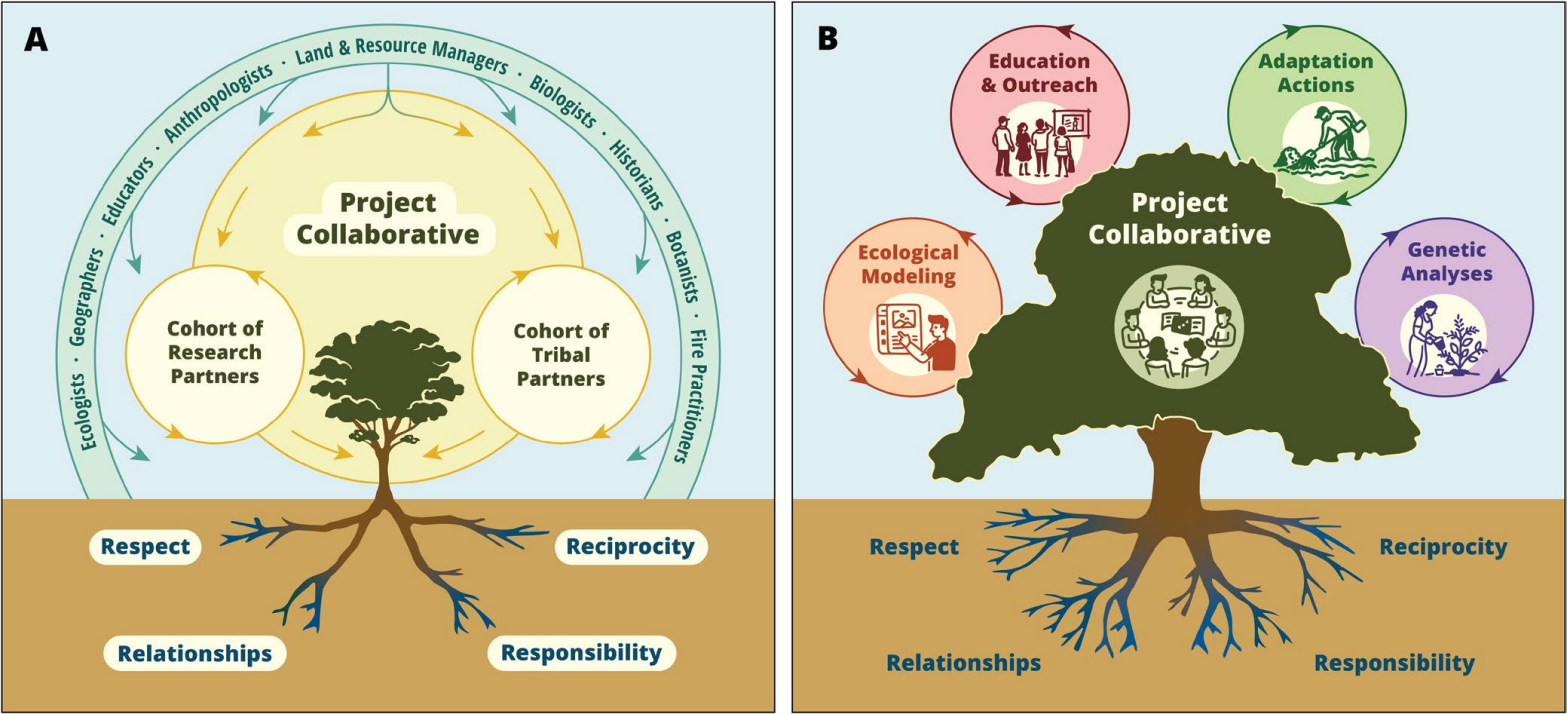
Conceptual framework for the Resilient Restoration Project. Panel A (left) shows the foundational relationship between Tribal partners and Research partners who worked together to guide the project. Potential beneficiaries of the work are depicted in the outer ring. Panel B (right) shows the outcomes that grew from the project collaborative from Panel A, starting with ecological modeling and genetic analysis that supported the development of adaptation actions and education and outreach.

The project, “Resilient Restoration: Advancing Ecological, Cultural, and Community Resilience with Tribal Nations in Southern California,” funded by the California Strategic Growth Council, was intentionally designed using a framework of Indigenous community‐led transformational climate adaptation (Figure 1). The framework was developed by the Climate Science Alliance (the Alliance; Pairis et al. 2023), a boundary‐spanning organization (Reed and Abernethy 2018) whose mission is to provide support for developing accessible information needed to adapt to climate impacts that are relevant at local and regional scales. The Alliance framework takes a transformational adaptation approach by centering community‐led activities that address communities' vulnerabilities to climate impacts, particularly with communities that have been historically and often intentionally excluded from conversations and planning efforts related to natural resources conservation and climate adaptation. The framework focuses on more than just science translation and strategy development; rather, it intentionally integrates culture and community into an iterative adaptation process (Pairis et al. 2023).

**FIGURE 2:**
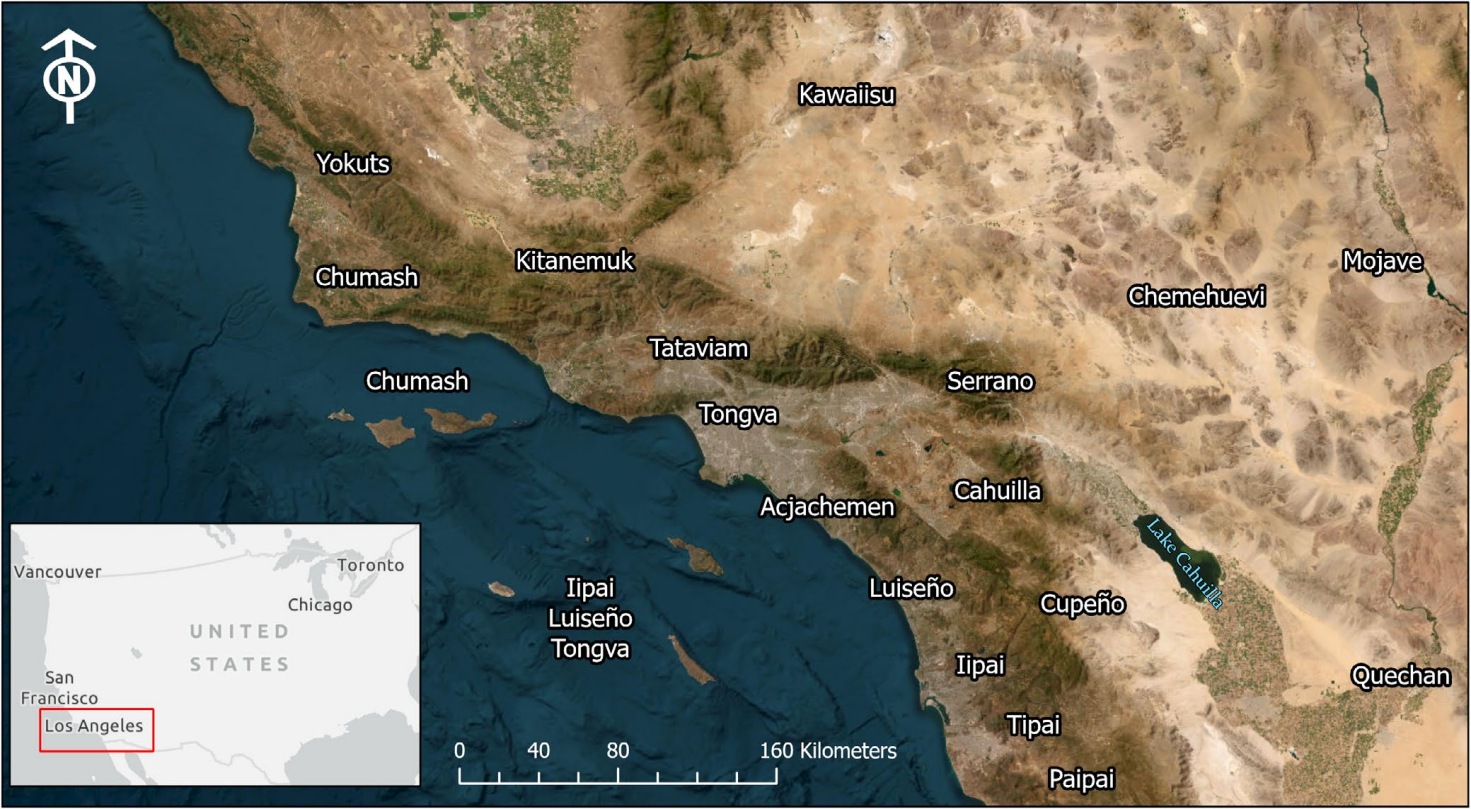
Regional context for the Resilient Restoration project showing Indigenous identities of Tribal communities associated with their ancestral lands in the coast, foothills, mountains and deserts of Southern California, USA, and northern Baja California Norte, Mexico. Inset shows geographic context of our study area, delineated by the red box. We have avoided delineating traditional use area boundaries, a contested concept.

The Alliance has implemented this process by convening working groups in which Indigenous peoples guide research goals and direct land stewardship practices supported by that research (Smith et al. 2023; Thoreson et al. 2024). The concept of the Resilient Restoration project was developed through the Alliance's Tribal Working Group, established in 2017, as an intertribal collaborative of more than 20 Tribal Nations and Indigenous organizations across southern California with ties across the southwest, including but not limited to Kumeyaay/Diegueño (Iipai, Tipai, Paipai), Payómkawichum/Luiseño, Cahuilla, Cupeño, Tongva, Tataviam, Serrano, Acjachemen, and Chumash peoples and their territories (Figure 2). To develop a vision for the project, the Tribal Working Group met monthly, facilitated by Alliance staff, with the participation of researchers who partnered with the Working Group to implement the Indigenous‐directed research in support of the group's goals. Together, participants discussed strategies for cooperation and collaboration to support the implementation of Traditional Ecological Knowledges (TEK or Indigenous knowledges; Whyte 2017) and land stewardship practices.

Underpinning our framework for Indigenous community‐led climate adaptation are authentic relationships with community partners based on *respect*, *responsibility*, and *reciprocity*. These core tenets were originally introduced as the ethical principles to guide efforts in higher education to address the unique needs and perspectives of Indigenous students (Kirkness and Barnhardt 1991) and have been explored and built upon by numerous other scholars. In particular, Tsosie et al. (2022) proposed including *relationships* for application of Indigenous research methodologies. Our collaborative work has embodied a collective commitment among all partners—Alliance staff, researchers, as well as Tribal community members—to these four principles.

Throughout the Resilient Restoration project, we expressed *respect* for Indigenous cultural practices and perspectives by engaging with each community on their own terms. We held meetings on Tribal lands, followed traditions and cultural norms, and took time to be in community with one another to plan the work. We sought input on research components of the project that adapted Western scientific research to include the Indigenous sciences and traditional practices community members were leading.

A *responsibility* towards knowledge sovereignty and Tribally led research is built into the Alliance's model, ensuring data and knowledges for and about Indigenous partners are used to advance community priorities and safeguard their lands and cultures from the threat of climate change (Pairis et al. 2023). Indigenous data sovereignty means Indigenous partners have the right to govern the collection, ownership, and application of their own data. In projects aimed at land stewardship and cultural revitalization, it is important that relationships between Indigenous and non‐Indigenous (research) partners are not extractive and that those relationships support ethical co‐creation between researchers and all others implicated in the work (Reid et al. 2024), as well as supporting Indigenous land stewardship goals (Thoreson et al. 2024). Following this model, the work of the Resilient Restoration project adhered to the CARE principles (Collective benefits; Authority to control; Responsibility; Ethics) for Indigenous data governance (Carroll et al. 2021). In practice, this meant that knowledge sovereignty principles were integrated and formalized into the project proposal, grant agreement, and collaboration agreements with Tribal partners from the outset.

As the project progressed, knowledge sharing and exchange were grounded in *reciprocity*. Tribal participants offered knowledges and perspectives to guide the work, which were then incorporated into different aspects of the project by Alliance team members and academic researchers, who would in turn offer up additional knowledges, information, and support for capacity building. We iterated through this process over the course of 4 years to develop a project that met the needs of Tribal community partners, reflected their perspectives and priorities, and allowed for Western science to contribute relevant information and support to advance Indigenous stewardship under shifting climatic conditions. Indigenous partners were compensated for their time as advisors and as leaders of activities and pilot projects that helped test and disseminate research findings.

Finally, to honor the *relationships* Indigenous peoples and communities have with the land and all living things, as well as the responsibility to both the ancestors and future generations, the work of the Resilient Restoration project followed the kincentric world view of the Tribal partners who participated (Silko 1997; Salmón 2000; Romero‐Briones et al. 2020). While Western science and academic research often focus on the importance of respect and reciprocity in the human relationships involved in research, it is also vital to recognize other living beings as kin or relatives and maintain respectful, reciprocal relationships with them. A lack of understanding or willingness to recognize Indigenous kin‐based worldviews and land‐ethics can damage collaborative relationships. In this project, Tribal partners identified the plant relatives they wanted to include in ecological modeling (Section 3.1) and shared their perspectives and stories about these relatives to ensure their reciprocal relationships were central to the work and upheld in project outcomes.

With these foundations in place, we embarked on an effort to bring together Western science and TEK to collaboratively vision stewardship of plant relatives under changing conditions into the future.

## 3 Resilient Ecological Restoration with Tribal Nations in Southern California

The Resilient Restoration project took place in the region now known as Southern California, its initial scope determined by the Tribal Working Group (Figure 2). Southern California includes part of the California Floristic Province and hosts some of California's most biologically diverse ecosystems (Jennings et al. 2018) that are important to Southwestern Tribal communities. In our collaboration, we focused on preserving terrestrial ecosystems and specific plants that are facing potential impacts of current and future climate change. The overarching goal was to integrate established research tools with traditional ecological and cultural knowledges to inform natural resource (natural relative) management (tending) practices and restoration efforts across the landscape under shifting climatic conditions. The project advanced Tribal resilience by supporting pilot projects to test restoration strategies and build and share capacity across Tribal communities.

While the numerous Tribal communities of this region share geography and kinship relationships, they are not monoliths and can exhibit complex governance structures within and across communities (Akins and Bauer 2021). Our project acknowledged these differences by working directly with participants from different communities to integrate their unique perspectives and be responsive to their comments and concerns. California's Indigenous peoples are known for their cultural and linguistic diversity and for tending the land with a level of intensity deemed noteworthy by those who study traditional lifeways (Anderson 2005, 34–40; Lightfoot and Parrish 2009). The history of colonialism in California is notable for its level of brutality (e.g., Mission slavery, extermination policies) and for leaving Tribes severely land poor (Akins and Bauer 2021). Consequently, the dispossession from their historical land base caused the academic gaze to shift towards seemingly isolated cultural traits, thereby obscuring the centrality of land stewardship and establishing a pattern of superficial, transactional inquiry. Southern California's Indigenous peoples have small or largely inaccessible reservations or none at all, and many Tribes lack federal recognition (Carrico 1987; Anderson 2005, 62–121; Mooney‐D'Arcy 2013; Chilcote 2024). This has resulted in distrust of contemporary institutions that want to work with Indigenous communities but are ignorant of this history. Academic researchers often see the relationship as purely transactional (Tuhiwai Smith 2012), do not create relationships to explore long‐term common goals, and fail to understand important differences and complexities across Tribal communities. To ensure our efforts were truly collaborative and dedicated to co‐creation with the range of Tribal communities across southern California, the project centered Tribal partners through the Tribal Working Group, which guided and informed the four major components of this project—Ecological Modeling, Genetic Analyses, Adaptation Actions, and Education & Outreach (Figure 1)—described in the following sections in the context of *respect*, *responsibility*, *reciprocity*, and *relationships*.

### 3.1 Ecological Modeling

To support Indigenous‐led stewardship planning and implementation under changing climatic conditions for plant relatives, academic scientists leveraged data and methods from contemporaneous work (Rose et al. 2023, 2024), developing habitat models under current and future climates for 50 culturally important plant species (Magee et al. under review). The models were generated using mapped environmental data including climate grids, as well as soils and topography. We compared the modeled distribution of species' current habitat and future habitat under climate change scenarios to identify habitat that is predicted to be stable (remains suitable) or become unsuitable (habitat loss), by both (consensus) or only one of the two climate models as a result of climate change.

We linked these habitat maps to demographic models that we created from data in published ecological studies and used them to predict plant population changes under different global change and restoration scenarios (Backus et al. 2025). Realistic population models require detailed data about species' growth, seed production, dispersal, and response to fire, as well as year‐to‐year variability in these parameters, which we were able to glean from previous studies or leverage from similar species. Population models were developed for a subset of the 50 species, including five oaks and four pines. Because most of these species have fire adaptations, we linked spatially explicit episodic fire events to the population models and accounted for changing fire regimes with future climate change (Syphard et al. 2024). These models demonstrated the importance of spatial patterns in fire frequency on plant population vulnerability (Backus et al. 2023). We simulated population dynamics under a range of climate and fire frequency scenarios.

**FIGURE 3:**
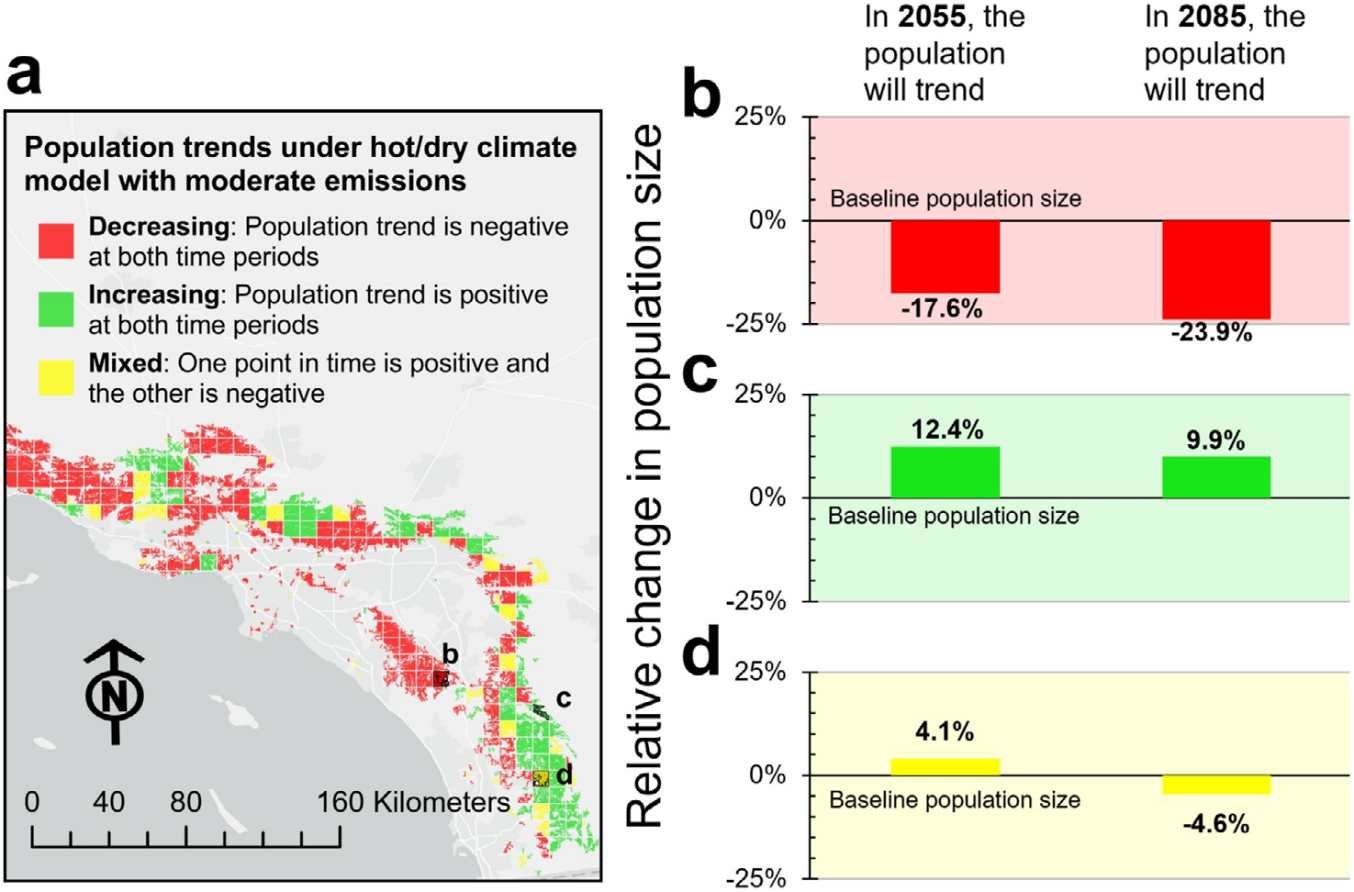
Example of ecological modeling products available in the Data Portal supporting climate adaptation and resilient restoration. (a) Spatially explicit population model projections for coast live oak ( *Quercus agrifolia* ) across southern California under the hot/dry climate model and moderate emissions scenario. (b–d) Three representative populations showing (b) a population expected to consistently decline for both modeled time steps (2055 and 2085), (c) a population expected to consistently increase, and (d) a population expected to both increase and decrease.

To provide an accessible platform for partners to explore the habitat and population models for stewardship and restoration activities (*relationships*, *reciprocity*), we developed a web‐based GIS‐based data portal (Figure 3). This access‐restricted Resilient Restoration Interactive Data Portal for Tribes was designed to archive important cultural information supporting climate adaptation efforts and cultural resilience (if desired), while also protecting sensitive information, honoring data sovereignty and knowledge sharing agreements determined by the Tribal Working Group (*responsibility*). We shared regular updates with partners at Tribal Working Group meetings and hosted four dedicated workshops to share data components and co‐produce the portal (*respect*, *relationships*). Together, we identified strategies for stewardship informed by the ecological modeling products that were integrated into an interactive adaptation menu to identify on‐the‐ground actions for restoration and resilience. Through this iterative process, we identified training needs to support Tribal use of spatial data, habitat maps, and population model output in decision making. We addressed those needs in the capacity‐building aspects of the project described below.

While constructing models and generating results, the ecological modelers attended monthly meetings to build *relationships* and seek feedback from Tribal partners on model input and output, and then revised models to better align with Tribal goals (*reciprocity*). In learning about principles of Indigenous knowledge sovereignty, researchers were struck by the realization that the current trend towards ‘open data and open science’ is a privilege of the dominant Western scientific tradition. We leveraged public or published data for the ecological models, but some information about plant species provided by the Tribal Working Group was not shared beyond the project and was retained in the access‐restricted Data Portal (*responsibility*).

Notably, the conversation about a prioritized list of culturally significant species was an illuminating one. Despite well‐established concepts of the interrelatedness of species in an ecosystem (e.g., Leopold 1949; Jones and Lawton 1995), it is nonetheless typical for conservation ecologists to ask participants in conservation planning efforts to prioritize species or other elements of biodiversity for conservation protection based on principles of extinction risk or functional or phylogenetic uniqueness (Bottrill et al. 2008; Regan et al. 2008). Furthermore, ethnobotanical studies often categorize plants according to their utilitarian uses (food, medicine, fiber, e.g., Bean and Saubel 1972, Native American Ethnobotany Database 2003 http://naeb.brit.org/), rather than their relationships to people or to each other. The concept that one plant relative should be prioritized over another for restoration planning purposes was, however, antithetical to the Working Group's concepts of *relationship* and *reciprocity* with nature. The research team learned to navigate new terrain in the research, *respecting* the ’focal species’ in simulation models as someone's relative, to whom we owe not just a scientific but a filial responsibility.

### 3.2 Genetic and Ecological Analysis of Oak Drought Resilience

To expand on the ecological modeling and more extensively address species‐specific concerns raised by Tribal partners, the academic research team focused on two oaks in the region, coast live oak ( *Quercus agrifolia* ) and Engelmann's oak ( *Q. engelmannii* ). Recognizing the importance of oak trees for Tribal partners, the team brainstormed possible metrics to track responses to drought stress such as mortality and growth as well as solutions that might increase the resilience of oak woodlands. One proposed approach was to assess genetic parameters to determine potential conservation practices. This assessment included genetic analysis of oak leaves to measure genetic diversity, differentiation, genetic structure of populations of oaks, and the extent of homozygosity which can reveal the expression of harmful deleterious alleles. Simultaneously, researchers proposed a greenhouse‐based common garden experiment to detect drought tolerant varieties, populations, and/or individuals. Following these discussions, research team members formalized partnerships with four Tribes through individual agreements to collaboratively sample acorns and oak leaves from their lands for genetic analysis and greenhouse experiments. We also collected samples from the ancestral lands of partners now managed by the US Department of Agriculture (USDA) Forest Service to expand geographic sampling. We processed ~4000 acorns, planted ~2500 oak seedlings in the greenhouse where watering experiments were used to measure plant performance such as germination, growth, stomatal conductance, and mortality under water‐stressed conditions.

**FIGURE 4:**
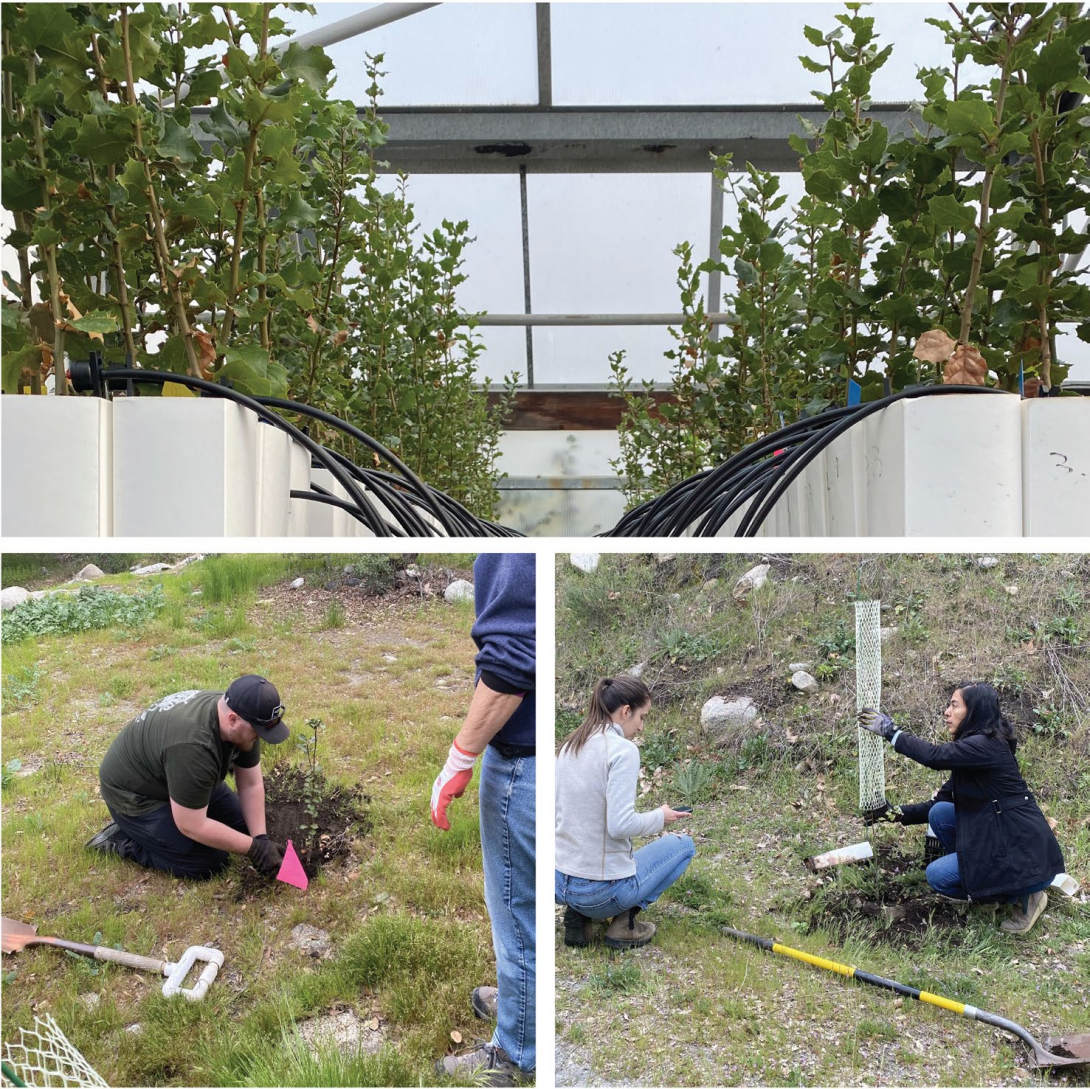
Rematriation of oak seedlings. Top: Seedlings from the greenhouse experiment, located in SDSU. Bottom: A community planting event on the La Jolla Indian Reservation where Tribal partners, community members, interns, researchers, and students collaborated to restore oak woodlands.

Once the greenhouse experiments were completed, all surviving plants were returned to Tribal partners for rematriation (*respect, reciprocity, relationships*). Rematriation signifies the restoration of a living entity to its rightful place on the land, emphasizing a spiritual connection and matrilineal perspective that is primarily used in Indigenous contexts (*relationships*). In addition, once the experiments were completed, all surviving plants were rematriated on Tribal lands (*reciprocity*) (Figure 4). Research team members and several Tribal partners collaboratively planned and hosted restoration events on Tribal lands, planting the rematriated seedlings in oak woodlands needing restoration. Seedlings were also given to Tribal members during community gatherings like Earth Day.

Before embarking on the genetic component of the research, it was important that research team members understood the history of harmful and extractive research on Tribal subjects. Tribal communities' concerns about extractive research are deeply rooted in a long history of scientific exploitation, racism, and cultural insensitivity. For example, in the Havasupai Tribe Case, Arizona State University researchers collected blood samples from the Havasupai people under the pretense of studying diabetes (Sterling 2011) but later used them without consent for studies on schizophrenia, migration patterns, and inbreeding, violating ethical agreements and causing cultural distress. To build a foundation of trust and address the harmful legacy of some scientific research, the research team met with the Tribal Working Group to clarify the study's intentions. A primary goal was to distinguish this research from other, more controversial types of genetic work. The researchers explained that the study would not involve genetic modification, where an organism's genome is altered. Tribal partners affirmed that such studies were not welcome. Instead, the project would use population genetics to estimate genetic diversity and population structure, which can assess oak health and resilience and inform conservation and management practices that benefit the species (Buck et al. 2023).

The issues Tribal partners raised during these discussions about genetic studies included risks of misrepresentation, exploitation, and loss of autonomy. There was an initial concern among Tribal partners about collection and development of genetic data for oaks as they are viewed as important relatives. Researchers and Tribal partners discussed both the value of and the concerns about the research focused on transparency, data sovereignty (*responsibility*), and protocols to ensure *respect* and care for these important relatives and ancestors (*relationships*). These discussions resulted in several key aspects of data collection and project management.

Overall, there was consensus about the advantages of having population genetic information to support Tribal development of management strategies for oaks. We collaboratively developed a framework for informed consent that explained the purpose and implications of the research on oaks. This framework included a benefit‐sharing agreement to establish how Tribal communities would gain from scientific advancements based on their genetic data (*reciprocity*). To ensure that all concerns about the collection, storage, and release of these data were appropriately managed (*responsibility*), the researchers confirmed in written agreements that Tribal partners would retain authority over the genetic data from their oak relatives. Genomic sequences have been protected by storing those data using coded identifiers to maintain confidentiality, and geographic coordinates are not publicly shared without the informed consent of Tribal partners. To enhance transparency and accountability (*respect*), Tribal members participated in field research and visited the greenhouse to engage in and learn more about the research process.

The oak research team also worked to foster Tribally led research. A protocol for acorn germination and oak propagation was created along with video demonstrations and shared with Tribal partners during pilot projects and training focused on plant propagation, including plant handling and storage. Cultural practices of *reciprocity* like asking permission of and giving thanks to land and plants were used during the collections on Tribal and non‐Tribal lands (*respect*). Seed handling was also considered. Seed surface sterilization is a crucial step in plant tissue culture, germination studies, and microbiological research to remove contaminants like fungi, bacteria, and spores from seed surfaces so they cannot be spread in the greenhouse or in common garden experiments. Sodium hypochlorite (NaOCl), the active ingredient in household bleach, is widely used in greenhouses, including Tribal nursery practices (Luna et al. 2009), because of its powerful oxidizing and antimicrobial properties. In fact, during acorn collection, university researchers discovered a novel pathogen affecting acorns that is currently under investigation. However, several Tribal partners on the project expressed that the use of bleach to surface sterilize oak seeds was unwelcome. Colorism and racial discrimination have played a role in how Indigenous peoples were treated and perceived by colonial powers (Brown et al. 2018). Native American communities faced forced assimilation policies, such as boarding schools and racial classification changes, and oral histories tell of bleaching of some Native Americans to lighten their skin. The use of alternatives to sodium hypochlorite for seed cleaning, such as hydrogen peroxide (Luna et al. 2009) or vinegar, has been proposed but their efficacy needs to be tested. Researchers on the team are now working with Tribal partners to develop culturally sensitive laboratory protocols that protect acorns against pathogens without the use of bleach.

### 3.3 Building and Sharing Capacity for Adaptation

To provide a connection between the research components described above and the stewardship practices and goals of Tribal partners, a Stewardship Pathways training program was established based on pilot projects to build and share capacity within and across Tribal communities *(reciprocity)* while allowing testing and demonstration of ecological restoration strategies and climate adaptation actions. Pilot projects included community programming to support sovereignty, cultural health/healing practices, and traditional teachings on wellness, replanting oak seedlings, and forest management through the revitalization of traditional and cultural uses of fire (Box 1). The Stewardship Pathways training program emerged from an explicit need expressed by the Tribal Working Group for hands‐on training opportunities to equip individuals—particularly within Tribal communities—with the knowledge and skills to use research findings to support their efforts to propagate native plants and advance climate‐resilient restoration projects. Rooted in the equal valuation of ways of knowing with an emphasis on the integration of climate and Indigenous science (Smith et al. 2023), the intent of the program is to build capacity, support economic and workforce development, and advance co‐stewardship of all ancestral lands. Two of the core Stewardship Pathways that were developed focused on native plant conservation, propagation, and restoration, and Indigenous fire stewardship (Box 2).

BOX 1 **Joelene Tamm (Squaxin Island Tribe), Natural Resource Director, La Jolla Band of Luiseño Indians.**

As a Tribal member from Washington State and a graduate student in entomology, I also serve as the natural resources director for a southern California Tribe that partnered with the Resilient Restoration project. This collaboration has allowed me to weave the strands of my life—my identity, academic research, and professional work—into a single, strong basket. This basket is now tight enough to hold the water of knowledge—both traditional and scientific—and carry it forward to heal the community and the land. I've been able to integrate my graduate research on the Goldspotted oak borer, an invasive pest devastating local oaks, with my work helping the Tribe rematriate fire to their ancestral lands.

Building on the network of relationships established by the project, this spirit of inquiry extended to our academic partners. We invited researchers to collect data on plant responses, soil health, and soil temperatures during the burn—a collaboration that unfolded spontaneously, driven by mutual trust. As a land manager, I know we must make research‐based decisions to move forward. By documenting the conditions that support a successful cultural burn, we can utilize all forms of science and knowledge, from academic inquiry to traditional practice, to make the most effective decisions for the land.

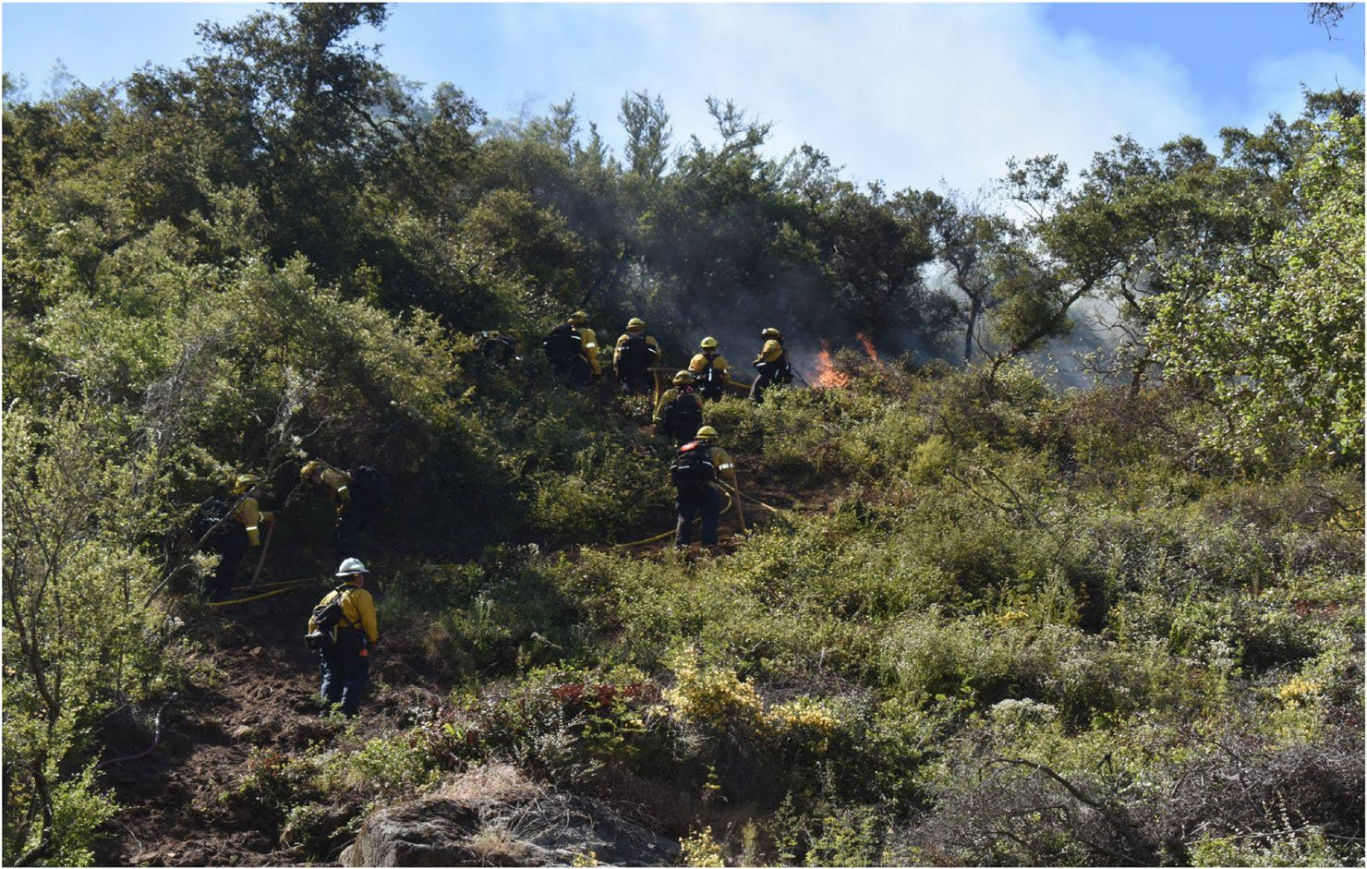

Fire crew facilitating a cultural burn held by the La Jolla Band of Luiseño Indians. Photo courtesy of La Jolla Band of Luiseno Indians.

BOX 2 **William Madrigal, Jr. (Cahuilla), Tribal Capacities and Partnerships Program Manager, Climate Science Alliance.**

A pilot project supported under the Fire Stewardship pathway on my Reservation was a cultural burn in a meadow to restore an important basket‐making plant. To re‐learn cultural burning practices, we brought a cultural practitioner, a relative from northern California, to articulate the importance of good fire in tending the land. The Resilient Restoration collaboration allowed us to expand our network, bring in allies with different expertise, and demonstrate that this practice of using good fire to heal the land could be successful. An initial site visit involved Tribal and agency partners to prepare for the Collaborative Burn. This Collaborative Burn supported the revitalization of cultural fire, the strengthening of interagency collaboration, and overcoming barriers and challenges to getting good fire back on the land. There is a current case study being generated to present the need for this as a climate benefit to the Tribe. It is the initial project of this kind for the Tribe and the Climate Science Alliance, with the hope that this will catapult similar joint collaborative burning throughout all partnering Tribes.

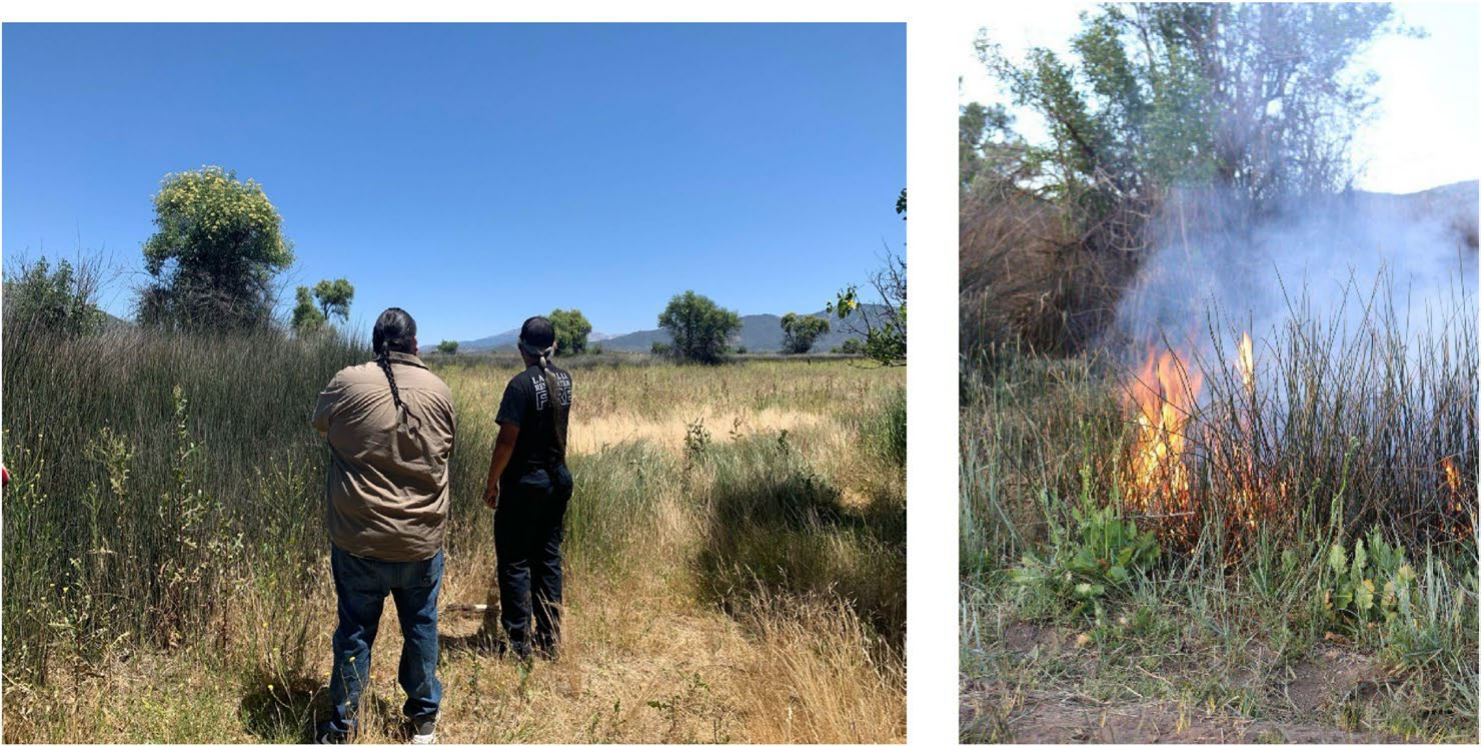

Left: Initial site visit to prepare for a collaborative burn event. Right: Burning in action.

For the plant restoration pathway, we designed monthly multi‐day training sessions focused on propagation techniques within a regional, climate‐informed, and cultural context. Trainees engaged in discussions with research scientists, Indigenous elders, and knowledge holders to learn about ethnobotanical uses of native plants and technical skills of plant collection and propagation. In subsequent sessions, trainees learned about forestry and restoration efforts on Tribal lands led by members of the Tribal Working Group and participated in restoration activities. Trainees visited a Tribally owned nursery to learn about nursery operations and practice techniques for processing and propagating seeds and bulbs. Participants also toured the facilities of a Tribal‐owned and operated organic farm where the values and the culture of the Tribe are integrated into the mission and operations of the farm, supporting their goal to create a sustainable lifestyle for the residents of their region. Participants engaged with elders and knowledge holders who taught about native plant species, plant pathogens, healthy soils, and programs focused on protecting native plants. These training events incorporated an intergenerational knowledge sharing approach that brought elders, youth, and community partners together with non‐Tribal researchers and practitioners.

A primary goal of the Indigenous fire stewardship pathway was to strengthen working relationships between Tribal communities and fire management agencies, including the federal USDA Forest Service and the State's CAL FIRE agency, and to foster collaboration between Tribal and non‐Tribal members (Box 3). Critically, the program focused on capacity building and sharing to reclaim and revitalize the cultural uses of fire—an exercise of Tribal sovereignty over a practice that was systematically suppressed by colonial authorities. The pathway consisted of several week‐long training events in topics such as basic wildland fire, resource advising, chainsaw safety, and weather. These trainings provided participants from across southern California with National Wildfire Coordinating Group (NWCG) certifications and foundational knowledge, successfully preparing them for careers in land management. One Tribal graduate now works as a full‐time wildland firefighter for the Forest Service, while a non‐Indigenous graduate was hired by the La Jolla Band of Luiseño Indians' Natural Resources Department. This training laid the foundation for future events to certify more wildland firefighters and Burn Bosses from Tribal communities, creating a pathway to restore cultural fire to the landscape. Graduates of this pathway supported the cultural burns described in Boxes 1–, 3.

BOX 3 **Clarissa Rodriguez, Postdoctoral Researcher, San Diego State University.**

As a postdoctoral researcher on the project and a plant ecologist studying trait plasticity in oaks, I was able to respond quickly to the invitation to collect data related to the cultural burn, facilitated by the collaborative spirit that guides the project and encourages support of Tribally determined research needs. I helped with the pre‐ and post‐fire vegetation surveys there to see what plant relatives are on the land, which ones come back with fire, which ones don't. Also, we are growing seeds from the soil bank in university greenhouses with the goal of returning germinants to their home to support revegetation. It was a gift to be present for the prescribed fire—I had studied fire ecology in the literature but had never been present at a cultural burn. To collaborate across scientific disciplines (with our narrow expertise—plants, soil, animals) and to bridge scientific and Indigenous knowledges required building relationships and trust, which takes time and effort, as well as good intentions.

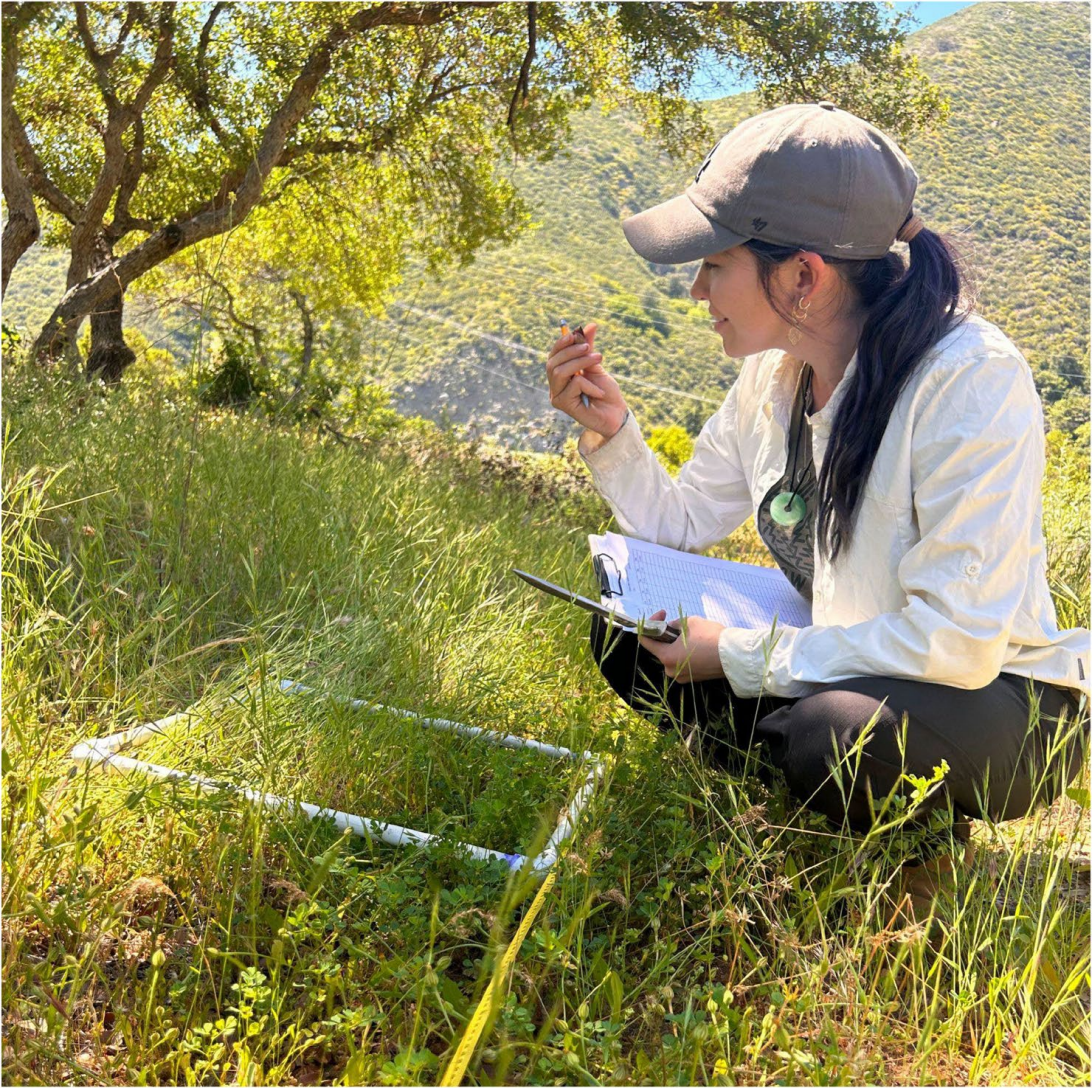

Clarissa Rodriguez gathering data as part of the pre‐burn vegetation survey.

These stewardship training programs offered opportunities for skill building and capacity sharing to accelerate adaptation through Indigenous stewardship. To advance knowledge sharing and collaboration more broadly for community action, we created a diverse range of opportunities for education and engagement.

### 3.4 Education and Engagement for Community Led Action

Education and engagement activities were developed at all phases of the project by coming together in community spaces, having conversations, sharing information, and listening (*respect, reciprocity*). This ongoing process allowed researchers to create community connections and build meaningful relationships by engaging Indigenous community partners in a variety of ways. From interactive hands‐on activities at community events to creating spaces that foster intergenerational knowledge exchange, the practice of continually showing up and creating unique opportunities to gather allowed us to collaboratively weave Indigenous knowledges and climate science directly into the research project. Thousands of hours were spent in community, participating in Earth Day events, providing input at workshops, offering stories and experiences at meetings and events, and sharing meals at informal gatherings *(reciprocity)*. This wide range of opportunities to connect as colleagues and friends fed directly into research efforts and the products that were tailored to serve communities.

**FIGURE 5:**
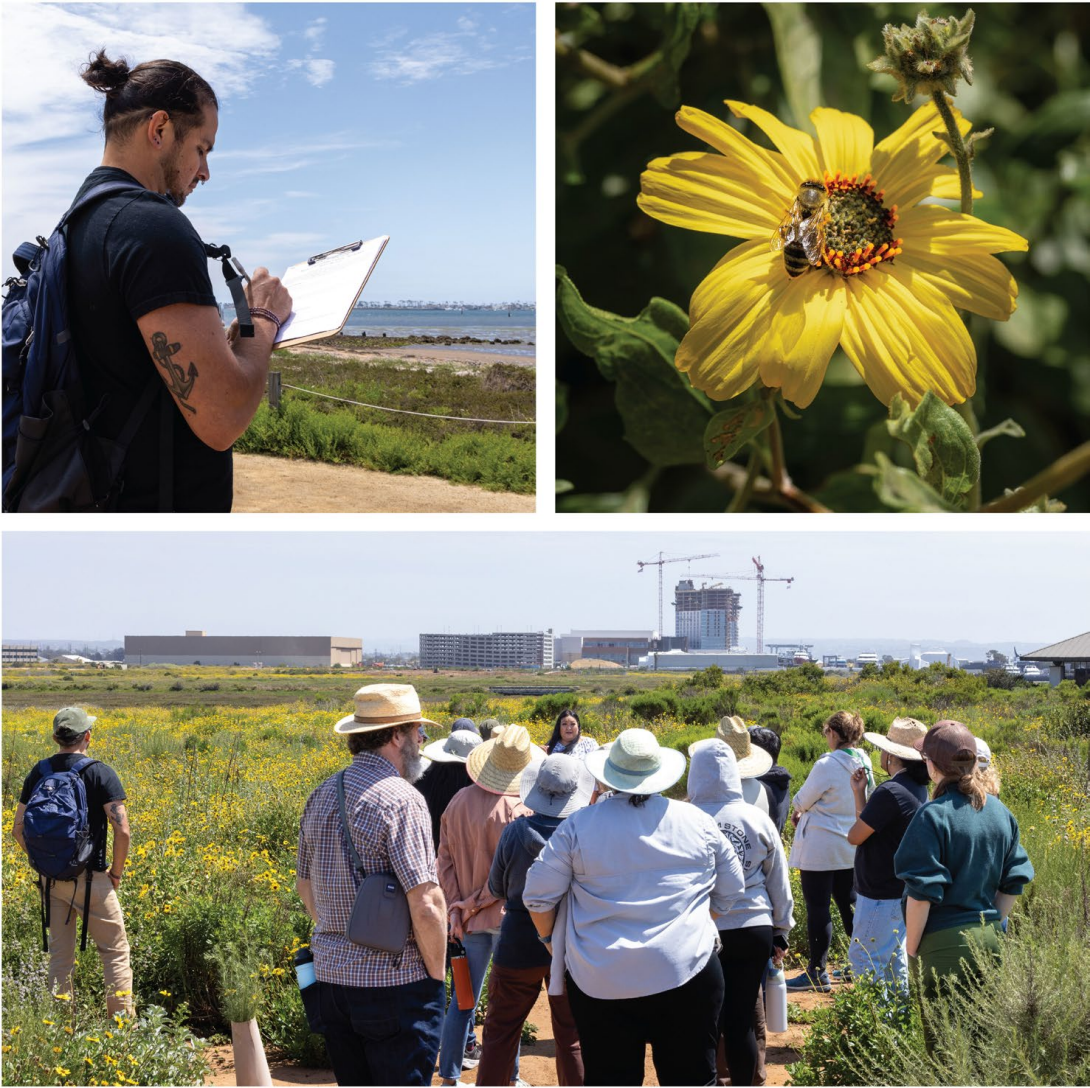
The second day of the training at Sweetwater Marsh. Photos by Condor Visual Media.

The culminating event for the project was a workshop designed to bring all the facets of the project together with a focus on sharing the co‐produced Resilient Restoration Data Portal (described in Section 3.1). The two‐day training, held on the ancestral lands of the Kumeyaay at the Sweetwater Marsh in southern San Diego County, California, provided time to explore the research outputs that were packaged in a user‐friendly format. Attendees heard from project contributors and explored the mapping components of the portal, discussing different stewardship scenarios where the information could be used. Day two of the training provided a deeper understanding of the history of the land and expected changes due to climate change with hands‐on activities. Attendees took to the field and practiced integrating information from the project with their own knowledges (Figure 5). This also provided a space to come together and reflect on the successes and future opportunities from a collaboration that resulted in stronger partnerships and more resilient restoration efforts to put adaptation into action through Indigenous stewardship.

## 4 Discussion

A deep commitment to building relationships based on respect, reciprocity, and responsibility has enabled a rich and successful collaboration among academic researchers, a boundary spanning organization, and Tribal land stewards in southern California. This work required a deliberate effort over the course of several years as well as acknowledgment of historical harms perpetrated on Indigenous communities through academic research, land/resource management, and conservation. Our collaborations have also required intentional efforts to reimagine how research endeavors are carried out in ways that differ from a typical academic model.

To build and maintain relationships for an effective collaboration, we started with *respect* for different cultural norms and Indigenous perspectives. For example, at the start of each group meeting or opportunity to come together, whether organized by the research team, the Alliance, or a Tribal partner, we opened with a blessing from an elder or culture bearer to start us off “in a good way.” This simple yet important acknowledgement of a common practice not only honored the cultures of our Tribal partners but also helped create a mindset and intention for all in attendance. Another critical perspective we embodied in the collaboration was respect for the people and the land and the fact that the two are inextricably linked. This respect carried forward into valuing different knowledge systems, for example, in workshops to review modeling results that explored ways of presenting results to be integrated with and/or complement TEK, depending on the preferences of project partners. During these conversations, we regularly talked about not only *what* partners needed, but also *how* they felt it could be most useful to them, acknowledging that the process of developing and implementing restoration with Indigenous knowledges is different from the Western scientific process (NASEM 2024). Prioritizing regular, open communication was critical to understanding differing perspectives and developing solutions that were respectful of all parties.

All our efforts to generate, synthesize, and disseminate data and information were governed by the researchers' *responsibility* to Tribal partners to protect data sovereignty. Our protocols followed existing guidance but navigating this in practice required flexibility. First and foremost, we honored the commitment that the information we produced was for use by the community and any distribution or sharing of that information should be determined by the community. However, in a diverse group of partners, we found that not all Tribal participants agreed on what was shared and how, which highlighted that data sovereignty may be viewed and carried out differently for different communities. It was important to approach the process with each partner individually and respect their wishes. Where there was information that required collective input and approval, we sought consensus. Where we could not reach consensus, we followed the wishes of those requesting the most restrictive controls on data or information. While the research team was fully committed to our responsibilities to uphold data sovereignty, it was not without challenges in an academic setting. The protection of Tribal data and input was written into the grant proposal to clarify how open data requirements could not universally apply without limitations. There was also concern from the universities about the potential for data sovereignty protocols to infringe on the researchers' legally protected right to publish project results. Complying with California State law required thoughtful conversation with university lawyers about the language we used to describe the limitations and clarification about our review process with Tribal partners for identifying data or information of concern and addressing it through redactions or removal of identifying information.

Our ongoing collaborations and efforts to identify and secure grants for the research to be implemented on the ground are one way the group has envisioned *reciprocity*. In the beginning, Tribal partners put their trust in the research team's commitment to appropriately use, protect, and return shared knowledges to support stewardship efforts. Along the way, this trust was reaffirmed as the researchers communicated regularly and worked to adapt the project to partner needs. Reciprocity was offered in other ways that are reflective of common cultural practices among Tribal communities. Tribal leaders and elders were asked for permission and guidance where appropriate. Those who shared cultural practices and provided blessings and knowledges were compensated for their time and expertise; cultural gifts and food were also offered. Most importantly, there were no expectations that our relationships would consistently be in equal balance. There was acceptance of an ebb and flow depending on the needs of the project and the partners involved. By being present and aware, particularly when non‐Tribal partners were invited to Tribal events or activities unrelated to the project, there was opportunity for deeper connection and ongoing thought about what else could be offered or shared.

Over the course of our collaboration, it was clear that the investment in *relationship* building and meaningful engagement that took place long before the project ever started was the true catalyst for advancing the impact and longevity of the research. Because trust had been built, it was easier to bring new research partners into an already developed collaborative framework where authentic co‐creation could take place. We recommend that long before a grant proposal is due, time and energy are invested in meeting with people and being present in the community in appropriate ways such as public events. Building trust with partners through capacity sharing and investing in a relationship that exceeds the boundaries of a single project or idea is critical to moving away from transactional research efforts. By listening and learning, one can collaboratively co‐develop goals, approaches, and projects that benefit the community alongside a research portfolio. In addition, *respecting* the *relationships* between Tribal partners and their plant and animal relatives was foundational to the work and essential for a successful collaboration. Taking the time to understand different perspectives and needs and setting clear intentions and processes for pursuing mutually agreed upon research projects in advance can open the door to more opportunities and formalized agreements with Tribal leadership that all parties can stand by. These partnerships can further our collective goals to build resilience and advance climate science and solutions driven by community values.

It is important to note that there is a greater level of *responsibility* in research where researchers are invited into community spaces to meet elders and family members, to hear stories and songs, and share meals prepared from the land. These types of relationships are not built during a grant proposal or even a single project. Instead, they are worthy of the time and thoughtfulness needed to change the historical model of academic research. Research with any community requires the researchers to be willing to approach new relationships and collaborative efforts with humility so they can be responsive, flexible, and adaptive. This is particularly true in work with Tribal communities where past research has extracted information and inflicted harm. The ancient adage of “listen twice, speak once” has served the researchers involved in this collaboration well and is valuable advice for others embarking on community‐led research.

The Resilient Restoration project has led to broader collaborations where Tribal partners are implementing Indigenous stewardship practices on the ground, informed in part by our collaboration. This is ultimately the goal of translational ecology (Enquist et al. 2017) and co‐creation of knowledge (Beier et al. 2017) but is more challenging to achieve when navigating the balance of different knowledge systems and overcoming systemic barriers to community‐led action. This collaboration gave us a tangible opportunity to support, with data science and computer modeling, distributional (McCauley et al. 2019) and reparational climate justice—equitable climate adaptation that goes beyond recognizing the disparate burden of climate change (recognitional justice), or consulting with front line communities (procedural), but supports the agency of overburdened communities to determine their own adaptation pathway. Our approach focused on bringing together Western science and Tribal knowledges and science because both knowledge systems have value in considering how society can manage and restore resilient landscapes, and the complement will give us a greater chance of accomplishing those goals in the face of a rapidly changing climate. The result is an experience that is not only transformational for the researchers but also an equitable and effective path towards transformational climate adaptation on a local, regional, and global scale.

## Author Contributions

**Megan K. Jennings:** conceptualization (lead), methodology (equal), project administration (equal), visualization (lead), writing – original draft (lead), writing – review and editing (equal). **Amber Pairis:** conceptualization (lead), methodology (equal), project administration (equal), visualization (lead), writing – original draft (lead), writing – review and editing (equal). **Althea Walker:** conceptualization (lead), methodology (equal), project administration (equal), visualization (lead), writing – original draft (lead), writing – review and editing (equal). **William Madrigal Jr.:** conceptualization (lead), methodology (equal), writing – original draft (lead), writing – review and editing (equal). **Diane Terry:** conceptualization (supporting), methodology (supporting), visualization (lead), writing – original draft (supporting), writing – review and editing (supporting). **Joelene Tamm:** conceptualization (supporting), methodology (supporting), writing – original draft (lead), writing – review and editing (equal). **Connor Magee:** conceptualization (lead), methodology (equal), writing – original draft (supporting), writing – review and editing (supporting). **Alexandra Hoff:** methodology (supporting), visualization (supporting), writing – original draft (supporting), writing – review and editing (supporting). **M. Brooke Rose:** methodology (supporting), visualization (supporting), writing – original draft (supporting), writing – review and editing (supporting). **Gregory A. Backus:** methodology (supporting), visualization (supporting), writing – original draft (supporting), writing – review and editing (supporting). **Clarissa Rodriguez:** methodology (supporting), visualization (supporting), writing – original draft (supporting), writing – review and editing (supporting). **Lluvia Flores‐Renteria:** conceptualization (supporting), methodology (supporting), project administration (supporting), visualization (supporting), writing – original draft (lead), writing – review and editing (equal). **Janet Franklin:** conceptualization (supporting), methodology (supporting), project administration (equal), visualization (supporting), writing – original draft (lead), writing – review and editing (equal). **Helen M. Regan:** conceptualization (supporting), methodology (supporting), project administration (equal), visualization (supporting), writing – original draft (lead), writing – review and editing (equal).

## Funding

This work was supported by the California Strategic Growth Council's Climate Change Research Program (Grant #CCR30009, Regan PI) with funds from California Climate Investments—Cap‐and‐Trade Dollars at Work, and the Collaborative of Native Nations for Climate Transformation & Stewardship (CNNCTS, Jennings PI) (The California Climate Action Seed and Matching Grants) UCOP grant#: R02CM708. Ecological modeling work was also supported by the National Science Foundation (1853697 Regan and Franklin PIs). Students supervised under Dr. Flores‐Renteria were supported also by USDA's National Institute of Food and Agriculture's “From Learning to Leading: Cultivating the Next Generation of Diverse Food and Agriculture Professionals Program” (NEXTGEN) grant no. 2023‐70440‐40156/project accession no. 1030734; and Hispanic Serving Institution Program grant no. 2022‐77040‐37620/project accession no. 1028695.

## Disclosure

*Positionality Statement*: The work described herein was conducted in a multicultural and multinational region influenced by the US and Mexico border and it is the result of a long‐term collaboration of self‐identified Indigenous (A.W., W.M. Jr., J.T., C.M., and L. F.‐R.), and non‐Indigenous scientists (M.K.J., A.P., D.T., A.H., M.B.R., G.A.B., C.R., J.F., and H.M.R.). A. Walker is Nez Perce, Hopi, and Gila River. W. Madrigal Jr. is Payómkawichum and Cahuilla and a member of the Cahuilla Tribe. J. Tamm is a member of the Squaxin Island Tribe and is the Director of Natural Resources for the La Jolla Band of Luiseño Indians. C. Magee is a citizen of the Pala Band of Mission Indians. L. Flores‐Renteria is Mēxihcātl, a member of the Koanalan Tlapakoyan and a Mexican immigrant in the United States. All co‐authors played a role in consulting and engaging with community members and staff from multiple Tribes across the region now known as southern California, described in Section 2 and Figure 2.

## Conflicts of Interest

The authors declare no conflicts of interest.

## Data Availability

We do not present analyses or data in this paper.
